# Bioaugmentation of a historically contaminated soil by polychlorinated biphenyls with *Lentinus tigrinus*

**DOI:** 10.1186/1475-2859-11-35

**Published:** 2012-03-23

**Authors:** Ermanno Federici, Mariangela Giubilei, Guglielmo Santi, Giulio Zanaroli, Andrea Negroni, Fabio Fava, Maurizio Petruccioli, Alessandro D'Annibale

**Affiliations:** 1 Department of Cellular and Environmental Biology, University of Perugia, Perugia, Italy; 2 Department for Innovation in Biological, Agro-Food and Forest Systems, University of Tuscia, Viterbo, Italy; 3 Department of Civil, Environmental and Materials Engineering, Unit of Environmental Biotechnology and Biorefinery, Alma Mater Studiorum, University of Bologna, Bologna, Italy

**Keywords:** Polychlorinated biphenyls, Lentinus tigrinus, Bioaugmentation, Soybean oil, Degradation, Dechlorination, Biodiversity, Microbial community structure

## Abstract

**Background:**

Several species belonging to the ecological group of white-rot basidiomycetes are able to bring about the remediation of matrices contaminated by a large variety of anthropic organic pollutants. Among them, polychlorobiphenyls (PCBs) are characterized by a high recalcitrance due to both their low bioavailability and the inability of natural microbial communities to degrade them at significant rates and extents. Objective of this study was to assess the impact of a maize stalk-immobilized *Lentinus tigrinus *CBS 577.79 inoculant combined with soybean oil (SO), as a possible PCB-mobilizing agent, on the bioremediation and resident microbiota of an actual Aroclor 1260 historically contaminated soil under unsaturated solid-phase conditions.

**Results:**

Best overall PCB depletions (33.6 ± 0.3%) and dechlorination (23.2 ± 1.3%) were found after 60 d incubation in the absence of SO where, however, the fungus appeared to exert adverse effects on both the growth of biphenyl- and chlorobenzoate-degrading bacteria and the abundance of genes coding for both biphenyl dioxygenase (*bph*) and catechol-2,3-dioxygenase. A significant (*P *< 0.001) linear inverse relationship between depletion yields and degree of chlorination was observed in both augmented and control microcosms in the absence of SO; conversely, this negative correlation was not evident in SO-amended microcosms where the additive inhibited the biodegradation of low chlorinated congeners. The presence of SO, in fact, resulted in lower abundances of both biphenyl-degrading bacteria and *bph*.

**Conclusions:**

The PCB depletion extents obtained in the presence of *L. tigrinus *are by far higher than those reported in other remediation studies conducted under unsaturated solid phase conditions on actual site soils historically contaminated by Aroclor 1260. These results suggest that the bioaugmentation strategy with the maize stalk-immobilized mycelium of this species might be promising in the reclamation of PCB-contaminated soils. The addition of SO to matrices contaminated by technical PCB mixtures, such as Aroclor 1242 and Delor 103 and characterized by a large preponderance of low chlorinated congeners, might not be advisable.

## Background

The ubiquitous contamination of terrestrial and aquatic ecosystems by polychlorinated biphenyls (PCBs) has become a matter of increasing concern due to their toxicity and ability to accumulate along the food chain [1]. PCBs theoretically encompass as many as 209 different compounds, generally referred to as congeners, with a number of chlorine substitutions in the biphenyl nucleus varying from 1 to 10. From their first production in the 1930s, until their ultimate ban in the 1990s, the overall PCBs production has been estimated to approximately amount to 1.3 million tons, significant aliquots of which have been released in the environment mainly due to either accidental spills or improper disposal [1]. The environmental persistence of PCBs is due to their high hydrophobicity and chemical stability and to the inability of natural aquatic and soil biota to perform their mineralization at a considerable rate [2]. While biphenyl and monochlorobiphenyls can serve as growth substrates for several aerobic bacterial species, the degradation of PCB congeners with more than one chlorine substituent occurs through co-metabolism in which biphenyl exerts the dual role of energy source and inducer of the PCB-degrading enzymes [2,3]. Such biphenyl-induced dioxygenase enzyme system is only able to attack congeners with a number of chlorine substituents varying from 1 to 5 and the effects of chlorination pattern on bacterial PCB degradation have been shown to be due to restrictions on 2,3- or 3,4-dioxygenase attacks [2].

Unlike bacteria, several white-rot basidiomycetes (WRB) have been shown to degrade technical PCB mixtures [4,5] in the absence of biphenyl, the use of which is considered impractical in bioremediation [6]. WRB possess an extracellular radical-based enzyme ligninolytic machinery with low substrate specificity and able to oxidize a wide array of persistent organic contaminants [7,8]. Although fungal extracellular phenoloxidases (EPO), such as laccase and Mn-dependent peroxidase, have been found to be unable to oxidize PCB congeners [9], they are able to perform the breakdown of some degradation intermediates such as their hydroxylated derivatives [10,11]. Similarly to bacteria, PCB degradation by WRB appears to decrease as the degree of chlorination increases [7,9]. However, any mechanistic interpretation on the effect of the chlorination pattern is prevented by the lack of information concerning the enzymatic basis of the fungal breakdown of these contaminants. The use of WRB in soil requires the addition of lignocellulosic wastes in order to improve their ability to compete with the resident microbiota [8,12]. Some of these wastes might be a valuable alternative to biphenyl addition due to their contents in either terpenes or phenylpropanoids which have a stimulatory effect on specialized PCB-degrading bacteria [13]. In this respect, maize stalks might be really valuable [14,15]. The reduced PCB bioavailability stemming from their high tendency to become adsorbed to organic matter colloids [16] has been shown to be partially counteracted by the use of synthetic [17] or biogenic [18,19] mobilizing agents (MAs).

Plant oils may be a valuable alternative to conventionally used MAs, due to their cost-effectiveness and ability in enhancing clean-up levels in soils contaminated by persistent organic pollutants [20,21]; to date, however, their use has not been reported in the remediation of PCB-contaminated matrices.

Thus, objective of this study was to assess the impact of a maize stalk-immobilized *Lentinus tigrinus *CBS 577.79 inoculant combined with soybean oil (SO) on both clean-up and resident microbiota of an actual Aroclor 1260-contaminated soil. To this aim, unsaturated solid-phase conditions were adopted owing to their similarity with those used at the field-scale remediation. On the one hand, the fungus was selected owing to its previously reported ability to degrade PCBs in the technical mixture Delor 106 [4] and their degradation intermediates (*i.e*., chlorinated benzoic acids) [22]. On the other hand, the above mentioned contaminated soil was chosen since a very limited PCB degradation had been previously therein observed after an aerobic biostimulation treatment in the presence of both biphenyl and randomly methylated-β-cyclodextrins [19]. Bioaugmented microcosms were compared with homologous incubation controls to highlight the impact of the fungus on: (i) microbial density of cultivable heterotrophic and specialized bacteria (ii) diversity of the indigenous bacteria and fungi, (iii) abundances of functional genes in PCB degradation and (iv) PCB degradation performances in the aged soil. These objectives were pursued by an integrated approach, consisting of a combination of specific chemical, microbiological and molecular methods.

## Results and discussion

### Time- and treatment-dependent evolution of fungal biomass and cultivable bacteria

**Figure 1:**
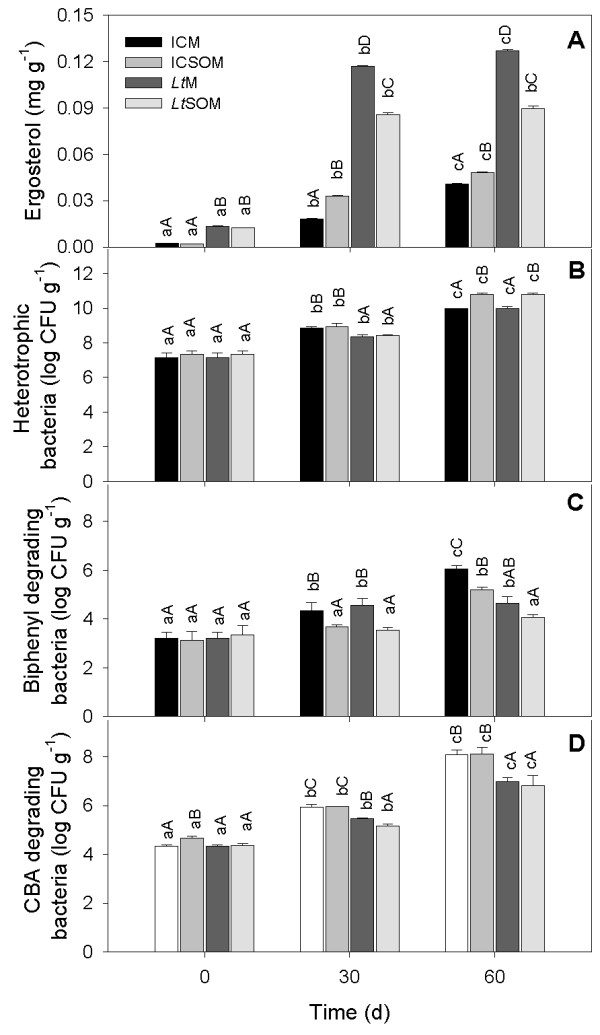
**Densities in heterotrophic and specialized bacteria and ergosterol contents in myco-augmented microcosms and respective controls**. Changes in concentration of indigenous cultivable aerobic bacteria and in ergosterol content after 0, 30 and 60 d treatment at 28°C in the incubation control and *Lentinus tigrinus *microcosms in the absence (*i.e*., ICM and *Lt*M respectively) and in the presence (*i.e*., ICSOM and *Lt*SOM) of soybean oil (2.5%, w/w). A, Total aerobic heterotrophic bacteria; B, specialized aerobic bacteria able to growth on biphenyl; C, specialized aerobic bacteria able to growth on monochlorobenzoic acids (CBA); D, ergosterol. Data are means ± standard deviations of 2 replicated experiments. Multiple pair-wise comparisons were performed by the Tukey test (P < 0.05). Same lower case and upper case letters denote absence of statistical significance between time-dependent changes within the same treatment and between treatments at the same time, respectively.

In the incubation control microcosms with and without soybean oil (ICM and ICSOM, respectively), the fungal biomass, indirectly estimated from the soil's ergosterol content, increased with time and the presence of soybean oil (SO) in the latter microcosm appeared to stimulate mycelial growth (Figure 1A). The fungal biomass also increased with time in *L. tigrinus *microcosm (*Lt*M) and was significantly higher than in non-bioaugmented microcosms. In the *L. tigrinus *microcosm with soybean oil (*Lt*SOM), instead, the biomass increased 7-fold within the early 30 d incubation to remain constant thereafter (Figure 1A). Regardless of the incubation time, fungal biomass in *Lt*SOM was lower than in *Lt*M.

Although the use of plant oils in mycoremediation has already been reported [20,21], very limited information is currently available on the impact of these additives on mycelial growth at soil moisture contents close to or lower than the water-holding capacity (WHC). In this respect, SO was found to exert a significant stimulatory action on the growth of several allochthonous fungi in soils spiked with polycyclic aromatic hydrocarbons [8,23].

The lower fungal biomass detected in *Lt*SOM than in *Lt*M might be ascribed to possible toxic effects exerted by SO-mobilized PCBs towards *L. tigrinus*. In addition, the significant amount of SO added to soil (*i.e*., 2.5%, w/w) might have resulted in a nutrient imbalance between added carbon and available nutrients thus increasing competitive effects between indigenous microbiota and *L. tigrinus*, as previously observed by Pizzul and collaborators [20].

Regardless of the presence or the absence of SO, *L. tigrinus *showed the ability to efficiently compete with the resident microbiota leading to ergosterol contents in *Lt*M and *Lt*SOM 6.5- and 2.6-fold higher, respectively, than in the corresponding incubation controls (*i.e*., ICM and ICSOM) (Figure 1A). The ability of *L. tigrinus *to antagonize indigenous microorganisms might be due to its reported ability to produce antimicrobial compounds able to inhibit a wide range of bacteria and fungi [24,25]. The inoculum formulation, using milled maize stalks (MMS) as the carrier, might be another determinant for the successful colonization of *L. tigrinus*. With this regard, several mycoremediation studies showed that lignocellulosic carriers confer to fungi either an initial competitive advantage over resident microbiota [26] or an increased tolerance to inhibitory effects exerted by contaminants [27].

The number of cultivable heterotrophic bacteria appeared to rise with time in all microcosms (Figure 1B). Pair-wise comparisons within the same incubation time of homologous microcosms (*i.e*., ICM *vs*. ICSOM and *Lt*M *vs. Lt*SOM) showed that their densities were not affected by the presence of SO after 30 d incubation and significantly enhanced in the successive harvest (Figure 1B).

A time-dependent growth increase of biphenyl-degrading bacteria (BDB) was only observed in ICM (Figure 1C). In fact, no significant changes in BDB densities were observed over time in *Lt*SOM while a delayed rise was evident in both *Lt*M and ICSOM (Figure 1C). Pair-wise comparisons within the same incubation times highlighted a depressive effect on BDB densities due to the presence of SO. With regard to the effect of the added fungus, significant differences between augmented *vs*. non-inoculated controls were only found at 60 d incubation where the former microcosms exhibited lower BDB concentrations. The concentrations of CBA-degrading bacteria, instead, tended to increase over time with no exceptions. However, in both 30- and 60-d-old microcosms, the densities of CBA degraders in *Lt*M and *Lt*-SOM were lower than in ICM and ICSOM.

### Evolution of bacterial and fungal community profiles and diversity

**Figure 2:**
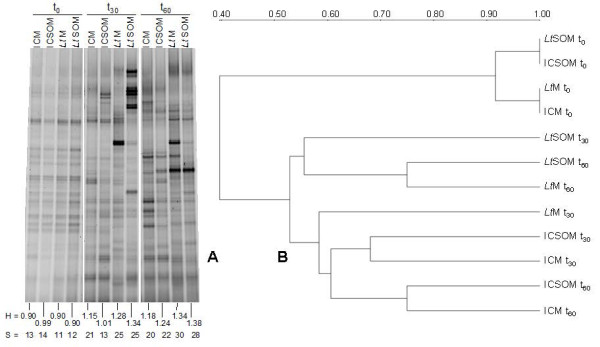
**DGGE analysis of the bacterial communities in myco-augmented microcosms and respective controls**. (A) DGGE analysis of the bacterial communities at start and after 30 and 60 d of incubation (t_0_, t_30 _and 60, respectively) at 28°C in the incubation control and *Lentinus tigrinus *microcosms in the absence (*i.e*., ICM and *Lt*M respectively) and in the presence (*i.e*., ICSOM and *Lt*SOM) of soybean oil (2.5%, w/w) and (B) cluster analysis obtained from the DGGE profiles based on the averaged similarity matrix. Scale indicates the degrees of similarity along of the nodes.

In order to gain more insights into the impact of both *L. tigrinus *and SO on the resident microbiota, a cultivation independent approach based on DGGE analysis of both 16S and 18S rRNA genes was employed taking into account that cultivable microbes represent only a minor fraction of the overall microbial community. DGGE analysis of 16S rRNA gene showed a time-dependent increase in both richness (*S*) and Shannon Weaver index (*H*) in all microcosms with the notable exception of the 30-d-old ICSOM where no significant changes in these parameters were found with respect to the zero time-point. The presence of *L. tigrinus *appeared to enhance the bacterial diversity as inferred by comparing bioaugmented microcosms with respective incubation controls. These results are in agreement with a previous mycoremediation study where the same fungal strain had been reported to improve the bacterial diversity [28]. Figure 2B shows the presence of two main clusters with the former including all microcosms at start. Within the latter cluster, bioaugmented microcosms segregated from non-inoculated ones with the exception of 30-d-old *Lt*M.

**Figure 3:**
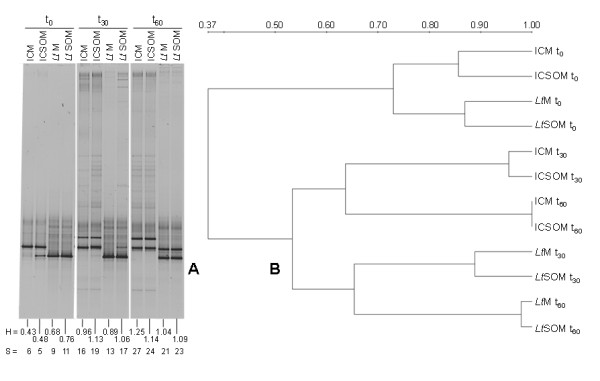
**DGGE analysis of the fungal communities in myco-augmented microcosms and respective controls**. (A) DGGE analysis of the fungal communities at start and after 30 and 60 d of incubation (t_0_, t_30 _and 60, respectively) at 28°C in the incubation control and *Lentinus tigrinus *microcosms in the absence (*i.e*., ICM and *Lt*M respectively) and in the presence (*i.e*., ICSOM and *Lt*SOM) of soybean oil (2.5%, w/w) (B) cluster analysis obtained from the DGGE profiles based on the averaged similarity matrix. Scale indicates the degrees of similarity along of the nodes.

DGGE analysis of 18S rRNA gene showed a poor biodiversity of the fungal biota at start with *S *and *H *values ranging from 5 to 11 and 0.43 to 0.76, respectively (Figure 3A). However, regardless of the microcosm type, both parameters appeared to increase over time. As opposed to bacteria, an increase in fungal diversity was already observed at the first harvest even in the presence of SO. Figure 3B shows the presence of two main clusters, the former including all the microcosms at start and the latter the remaining harvests. Within the latter one, bioaugmented microcosms displayed a low level of similarity (*i.e*., 0.53) with the non-inoculated ones. Low levels of biodiversity of the mycobiota, a common pattern in historically contaminated soils [29], had been also observed by Tigini et al. [30] in another Aroclor 1260 contaminated soil and ascribed to the selective pressure on the resident fungal community due to the concomitant presence of PCBs and heavy metals. As opposed to Tigini et al. [30], who observed lower fungal loads and species number after 120 d of either biostimulation or bioaugmentation of that soil, both fungal density and biodiversity increased regardless of the presence or the absence of the exogenous fungus. However, those findings were limited to cultivable mycobiota [30].

### Functional aspects of resident microbial community in PCB-contaminated microcosms

**Figure 4:**
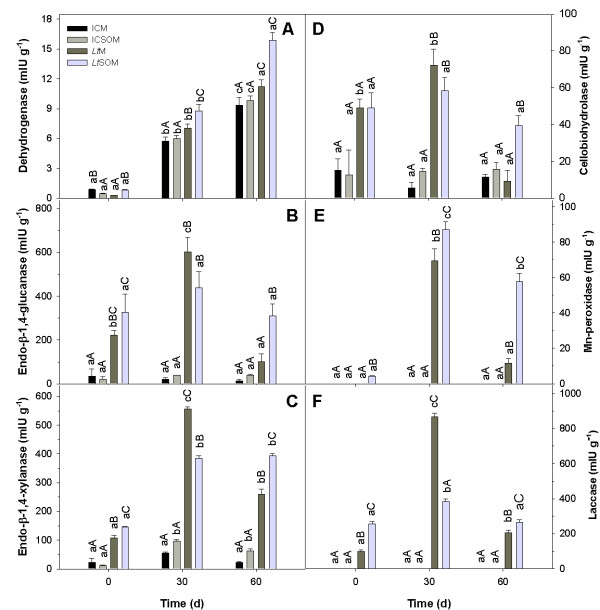
**Dehydrogenase, extracellular glycosyl hydrolase and phenoloxidases activities in myco-augmented microcosms and respective controls**. Dehydrogenase (A), extracellular glycosyl hydrolase (B-D) and phenoloxidase (E-F) activities at start and after 30 and 60 d of incubation at 28°C in the incubation control and *Lentinus tigrinus *microcosms in the absence (*i.e*., ICM and *Lt*M respectively) and in the presence of 2.5% (w/w) soybean oil (*i.e*., ICSOM and *Lt*SOM). A, dehydrogenase; B, endo-β-1,4-glucanase; C, endo-β-1,4-xylanase; D, cellobiohydrolase; E, manganese-peroxidase; F, laccase. Data are means ± standard deviations of two replicated experiments. Multiple pair-wise comparisons were performed by the Tukey test (P < 0.05). Same lower case and upper case letters denote absence of statistical significance between time-dependent changes within the same treatment and between treatments at the same time, respectively.

In all microcosms, dehydrogenase activity appeared to increase with the incubation time (Figure 4A). However, at both harvests, this activity was strikingly higher in bioaugmented microcosms than in non-inoculated ones; in the former and in the latter microcosms, the presence of SO either increased or did not affect dehydrogenase activity, respectively (Figure 4A). Dehydrogenase activity has been shown to be a valuable and robust indicator of detoxification in contaminated soils [31]. However, in this study, it was not significantly correlated with residual PCB contents in all the microcosms (R^2 ^= 0.184; *P *= 0.29) although its time-dependent rise denoted an increased microbial activity.

All the microcosms shared the presence of MMS mainly composed by cell wall polysaccharides and lignin [32]. Thus, the time courses of extracellular glycosyl-hydrolase (EGH) and extracellular phenoloxidase (EPO) activities were determined as additional indices of metabolic activity in the investigated microcosms. In non-bioaugmented microcosms, despite the observed time-dependent increase in fungal biomass, EGH activities did not significantly vary over time with the only exception of endo-1,4-xylanase in the ICSOM; in the same microcosms, EPO activities were not detected (Figure 4C).

In *Lt*M, conversely, all the EGH and EPO activities exhibited similar trends characterized by an initial increase after 30 d followed by a significant decline in the subsequent harvest. The presence of SO in *Lt*SOM appeared to mitigate the aforementioned activity decline of endo-β-1,4-glucanase, endo-β-1,4-xylanase and cellobiohydrolase in the 30-60 d time range (Figures 4B, C and 4D, respectively); the same trend was also observed for both laccase and Mn-dependent peroxidase activities (Figures 4E and 4F, respectively).

**Figure 5:**
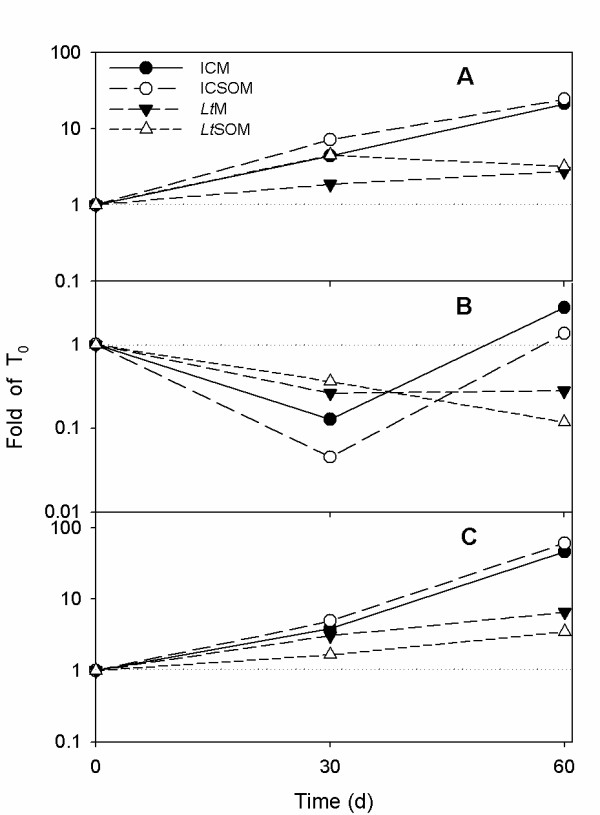
**q-PCR analyses of *16S rRNA*, *bph *and *C230 *abundances in myco-augmented microcosms and respective controls**. Semi-logarithmic plots of time dependent changes in the relative amount of *16S rRNA *(A) *bph *(B), *C230 *(C) qPCR-amplified genes in the incubation control and *Lentinus tigrinus *microcosms in the absence (*i.e*., ICM and *Lt*M respectively) and in the presence of 2.5% (w/w) soybean oil (*i.e*., ICSOM and *Lt*SOM). Data are expressed as fold with respect to the relative zero time-point (please, see Materials and Methods).

The abundances of the 16S rRNA gene in ICM and ICSOM increased approx. 21- and 24-fold, respectively, with respect to the zero time point after 60 d incubation (Figure 5A). Albeit to a lesser extent, such an increase was also observed in bioaugmented microcosms (*i.e*., approx. 2.8 and 3.2-fold in *Lt*M and *Lt*SOM, respectively).

The degradation of low chlorinated PCBs by aerobic bacteria is often initiated by a biphenyl 2,3-dioxygenase (bph), belonging to the family of Rieske non-heme iron oxygenases, which catalyzes the incorporation of molecular oxygen at the 2,3 position of the non-chlorinated or lesser chlorinated ring of PCB to form *cis*-dihydrodiol compounds [2,6]. The time course of biphenyl dioxygenase (bph) gene abundances in IC and ICSOM showed a marked decline after 30 d followed by a slight increase at 60 d (2.8- and 1.3-fold, respectively). In bioaugmented microcosms, *bph *abundances, instead, were lower than in the same microcosms at start regardless of the incubation time (Figure 5B) thus suggesting a competitive activity exerted by the fungus and confirming the BDB enumeration results (Figure 1C).

The aerobic conversion of chlorobenzoates, common PCB degradation intermediates, usually proceeds *via *the formation of chlorinated catechols, which, in turn, undergo either *ortho*- or *meta*-cleavage [2,3]. The latter ring fission mechanism is brought about by 2,3-catechol-dioxygenase yielding hydroxy-substituted chloromuconic semialdehydes [2]. With regard to the abundance of the 2,3-catechol-dioxygenase (C230) gene, pertaining to the lower PCB degradation pathway, a time-dependent increase was found for all microcosms although the highest folds were displayed by the non-bioaugmented ones (46- and 61-fold for ICM and ICSOM, respectively) (Figure 5C). The presence of SO appeared to exert an opposite impact on C230 gene, the abundance of which was positively and negatively affected in the incubation controls and the augmented microcosms, respectively (Figure 5C).

### PCB degradation

Table 1 shows both identities and respective initial concentrations of PCB congeners in ICM and ICSOM, the overall contents of which were higher than 700 mg kg^-1^. Three- up to hepta-chlorinated CBs encompassed the large majority of contaminants therein detected and, among them, the largest relative abundances were observed for tetra- and hexa-CBs (about 26 and 23%, respectively). PCB recoveries preliminarily investigated both in the absence and in the presence of MMS, which was employed in all microcosms, did not significantly differ. With few exceptions, the highest percent depletions of di-, tri-, tetra- and penta-CBs were observed in *Lt*M that also exhibited better removal performances towards hexa- hepta and octa-CBs than its corresponding incubation control (*i.e*., ICM). Despite these differences, a highly significant negative correlation between contaminant removal and degree of chlorination was found in both ICM and *Lt*M (R^2^adj equal to 0.968 and 0.981, respectively, *P *< 0.001). In this respect, it is known that lipophilicity and molar water solubility of PCBs tend to increase and decrease, respectively, as the degree of halogenation increases [33] and, thus, high chlorinated congeners show a low susceptibility to biodegradation [2,3].

**Table 1:** Initial PCB congeners concentrations and percent degradation in myco-augmented and control microcosms

Target soil PCBs (congener)	Initial concentration (mg kg^-1 ^soil)		Average depletion (%)*			
	
	Soil as such	Soil with SO	ICM	ICSOM	*Lt*M	*Lt*SOM
2,6-/2,2'-CB	1.35 ± 0.004	1.05 ± 0.003	85.6c	0.0a	44.6b	0.0a
2,4-/2,5-CB	0.09 ± 0.001	0.12 ± 0.003	94.3b	16.2a	37.2a	21.7a
2,3'-CB	0.43 ± 0.001	0.35 ± 0.003	0.0a	36.1b	89.1c	33.7b
2,4'-/2,3-CB	0.11 ± 0.001	0.08 ± 0.001	100b	39.5a	82.7b	47.3a
2,2',6-CB	2.73 ± 0.000	2.62 ± 0.004	37.9b	23.2a	59.4c	23.9a
2,2',5-/2,2',4-/4,4'-CB	39.33 ± 0.053	36.15 ± 0.097	44.2b	23.9a	52.9c	25.4a
2,3,6-/2,3',6-CB	3.07 ± 0.000	2.87 ± 0.009	42.7b	24.8a	50.8c	27.0a
2,2',3-/2,4',6-CB	16.14 ± 0.018	14.99 ± 0.045	41.0b	25.3a	50.1c	27.3a
2',3,5-CB	0.23 ± 0.001	0.21 ± 0.002	48.0b	29.1a	45.6b	31.8a
2,4,5-CB	0.21 ± 0.001	0.18 ± 0.000	48.7c	26.5a	51.9d	29.5b
2,3',5-CB	7.29 ± 0.007	6.66 ± 0.019	39.2c	25.2a	43.1d	28.9b
2,3',4-CB	2.28 ± 0.007	2.04 ± 0.004	38.9c	23.6a	43.3d	26.0b
2,4',5-/2,4,4'-CB	60.03 ± 0.111	55.17 ± 0.157	37.7c	25.8a	40.8d	29.1b
2,3,3'-/2',3,4-/2,2',5,6'-CB	7.45 ± 0.009	6.77 ± 0.019	36.7c	23.7a	42.9d	26.7b
2,2',4,6'-/2,3,4'-CB	27.83 ± 0.041	25.60 ± 0.070	34.9c	26.1a	41.6d	29.3b
2,2',3,6-CB	6.57 ± 0.008	6.15 ± 0.017	37.6b	27.1a	47.2c	29.8a
2,2',3,6'-CB	3.09 ± 0.001	2.90 ± 0.009	35.4b	30.5a	49.9c	31.5a
2,2',5,5'-CB	26.04 ± 0.035	24.31 ± 0.063	35.9c	26.3a	39.8d	29.9b
2,2',4,5'-CB	18.55 ± 0.022	17.24 ± 0.065	36.0c	26.6a	40.0d	30.1b
2,2',4,4'-/2,2',4,5-/2,4,4',6-CB	9.55 ± 0.018	8.83 ± 0.010	36.5c	26.8a	42.8d	30.3b
3,3',4-CB	0.85 ± 0.002	0.79 ± 0.006	30.7ab	27.4a	36.8b	32.8b
2,2',3,5'-CB	18.83 ± 0.039	17.44 ± 0.034	34.2c	25.3a	38.3d	29.2b
3,4,4'-/2,3,3',6-/2,2',3,4'-CB	10.38 ± 0.013	9.54 ± 0.032	31.7c	26.9a	38.2d	30.1b
2,2',3,4-/2,3,4',6-CB	20.96 ± 0.037	19.39 ± 0.055	33.2c	27.9a	39.8d	31.9b
2,2',3,3'-CB	6.01 ± 0.006	5.56 ± 0.017	31.1c	26.6a	40.6d	29.8b
2,3',4,5-CB	2.45 ± 0.001	2.39 ± 0.008	28.9a	28.6a	35.5c	32.6b
2,3,4',5-CB	1.23 ± 0.001	1.11 ± 0.003	28.1b	26.0a	29.6b	28.9b
2,4,4',5-CB	12.83 ± 0.011	12.07 ± 0.045	30.2b	27.6a	33.3d	31.0c
2,3',4',5-CB	21.95 ± 0.030	20.60 ± 0.071	29.6b	27.5a	34.1d	30.7c
2,2',3,5',6-CB	25.58 ± 0.049	24.07 ± 0.070	28.2a	25.4b	33.1c	28.0a
2,2',3,4',6-CB	1.20 ± 0.001	1.15 ± 0.003	30.5a	31.0a	32.0a	32.4ab
2,3,4,4'-/2,3,3',4'-CB	18.78 ± 0.032	17.44 ± 0.051	28.3a	27.4a	34.2c	30.1b
2,2',3,5,5'-CB	4.42 ± 0.005	4.25 ± 0.022	27.0a	25.5a	30.6c	28.6b
2,2',3,3',6-CB	8.47 ± 0.032	7.92 ± 0.022	31.0b	28.0a	36.9c	30.3b
2,2',3,4',5/2,2',4,5,5'-CB	30.81 ± 0.052	29.22 ± 0.040	25.2a	24.6a	30.6c	26.9b
2,2',4,4',5-CB	7.49 ± 0.013	7.34 ± 0.004	28.6a	30.0b	32.2c	32.1c
2,3',4,4',6-CB	0.33 ± 0.002	0.23 ± 0.184	28.1b	0.1a	0.3a	0.0a
2,2',3,3',5-CB	1.29 ± 0.016	1.36 ± 0.006	28.5b	29.0b	16.2a	32.0c
2,2',3',4,5-CB	6.77 ± 0.002	6.44 ± 0.008	28.7b	25.2a	31.2c	28.9b
2,2',3,4,5'/2,3,4,4',6-CB	12.10 ± 0.008	11.40 ± 0.017	27.2a	27.0a	31.7c	29.8b
2,2',3,4,4'-CB	3.09 ± 0.002	3.06 ± 0.052	25.7a	30.9b	29.8b	37.3c
2,2',3,3',6,6'-CB	8.43 ± 0.017	8.01 ± 0.042	23.5a	22.3a	28.3b	24.4a
2,3,3',4',6-CB	19.87 ± 0.010	18.75 ± 0.010	25.6a	25.7a	30.5c	27.9b
2,2',3,5,5',6-CB	11.81 ± 0.008	11.30 ± 0.023	21.2a	22.1a	26.4c	23.9b
2,2',3,3',5,6'-CB	8.85 ± 0.008	8.47 ± 0.016	21.3a	22.6a	25.1b	24.2b
2,3,3',4',5-CB	0.91 ± 0.000	0.91 ± 0.009	26.3a	26.3a	54.0b	31.2b
2,2',3,4',5',6/2,3',4,4',5-CB	34.83 ± 0.024	33.30 ± 0.067	22.0a	23.5b	27.2d	25.3c
2,2',3,3',5,6-CB	2.94 ± 0.007	2.79 ± 0.006	22.5a	23.4a	28.2c	25.2b
2,2',3,3',4,6/2',3,3',4,5-CB	2.60 ± 0.002	2.51 ± 0.007	21.7a	24.2b	29.1d	26.8c
2,2',3,4',5,5'-CB	5.51 ± 0.005	5.38 ± 0.011	20.1a	24.3b	26.7c	26.1c
2,2',3,3',4,6'/2,2',4,4',5,5'/2,3,3',4,4'-CB	60.87 ± 0.069	58.57 ± 0.145	20.3a	23.4b	26.6c	25.1c
2,2',3,4,5,5'/2,2',3,3',5,6,6'-CB	13.51 ± 0.019	12.99 ± 0.033	18.8a	21.6b	24.7c	23.3c
2,2',3,3',4,5' + 2,2',3,3',4,6,6'/2,2',3,4,4',5-CB	3.92 ± 0.007	3.79 ± 0.012	18.4a	21.6b	24.9c	23.4c
2,3,3',4,5,6/2,2',3,4,4',5'/2,3,3',4,4',6-CB	34.54 ± 0.088	33.22 ± 0.137	20.6a	23.9b	26.8c	25.6c
2,2',3,3',4,5/2,2',3,3',5,5',6-CB	6.67 ± 0.002	6.48 ± 0.009	13.2a	17.9b	20.9d	19.3c
2,2',3,3',4,5',6 + 2,2',3,4',5,5',6-CB	10.52 ± 0.020	10.26 ± 0.036	15.4a	19.9b	22.2b	21.3b
2,2',3,3',4',6,6'-CB	4.89 ± 0.009	4.76 ± 0.016	16.8a	21.1b	20.4c	22.6c
2,2',3,4,4',5',6 + 2,2',3,3',4',4' + 2,3',4,4',5,5'-CB	9.87 ± 0.012	9.54 ± 0.040	20.6a	24.5b	27.0c	26.5c
2,2',3,4,5,5',6-CB	3.15 ± 0.002	3.08 ± 0.011	14.8a	19.6b	21.0b	21.2b
2,2',3,3',4,5,6'-CB	10.35 ± 0.021	10.06 ± 0.027	16.0a	20.4b	22.7b	21.8b
2,2',3,3',4',5,6-CB	5.92 ± 0.012	5.76 ± 0.019	16.0a	20.7b	22.8d	22.1c
2,2',3,3',5,5',6,6'/2,2',3,3',4,4',6/2,3,3',4,4',5-CB	10.61 ± 0.014	10.70 ± 0.040	16.0a	23.2c	22.0b	24.9d
2,2',3,3',5,5',6,6'/2,3,3',4,4',5'/2,2',3,3',4,5',6,6'-CB	3.73 ± 0.012	3.63 ± 0.016	12.1a	17.6b	20.4d	19.2c
2,2',3,3',4,5,5' + 2,2',3,3',4,4',6,6'-CB	2.30 ± 0.003	2.08 ± 0.012	20.1a	19.4a	27.6c	21.0b
2,2',3,4,4',5,5' + 2,3,3',4',5,5',6-CB	22.51 ± 0.043	22.07 ± 0.075	15.9a	21.4b	23.3c	23.0c
2,3,3',4,4',5',6-CB	0.82 ± 0.006	0.84 ± 0.009	15.2a	22.2b	16.6a	23.2b
2,2',3,3',4,5,6,6'-CB	0.91 ± 0.001	0.89 ± 0.003	8.3a	14.9b	12.6c	16.2c
3,3',4,4',5,5'-CB	0.11 ± 0.000	0.08 ± 0.001	26.1a	25.9a	39.3b	26.6a
2,2',3,3',4,4',5/2,3,3',4,4',5,6-CB	14.21 ± 0.029	13.90 ± 0.047	15.6a	21.0b	22.9c	22.7c
2,2',3,3',4,5,5',6-CB	0.21 ± 0.00	0.21 ± 0.001	35.1a	41.4b	42.3b	41.8b
2,2',3,3',4,5,5',6 + 2,2',3,3',4,5,5',6'	2.82 ± 0.003	2.77 ± 0.008	13.1a	19.1b	19.7b	22.8c
2,2',3,4,4',5,5',6/2,2',3,3',4,4',5',6-CB	3.82 ± 0.002	3.79 ± 0.020	12.8a	19.5b	19.7b	23.1c
2,3,3',4,4',5,5'-CB	0.34 ± 0.001	0.34 ± 0.000	16.3a	22.0b	24.1c	24.6c
2,2',3,3',4,5,5',6,6'/2,2',3,3',4,4',5,6-CB	1.82 ± 0.001	1.79 ± 0.005	12.0a	18.1b	20.0b	19.7b
2,2',3,3',4,4',5,6,6'-CB	0.11 ± 0.000	0.11 ± 0.000	10.3c	13.2b	11.5a	14.8c
2,2',3,3',4,4',5,5'-CB	2.97 ± 0.005	2.96 ± 0.011	13.0a	19.8b	16.7b	21.9c
2,3,3',4,4',5,5',6-CB	0.29 ± 0.011	0.26 ± 0.000	16.8c	14.2b	21.3d	21.1a
2,2',3,3',4,4',5,5',6-CB	0.93 ± 0.052	0.91 ± 0.064	0.0a	0.0a	0.0a	5.1b
Overall PCBs	776.3 ± 1.2	733.9 ± 2.3	28.0	24.5	33.8	26.9

Initial PCB congeners concentrations in maize stalk-amended soil in the absence (Soil as such) and in the presence of soybean oil (Soil with SO) and removals (%) in the respective controls (*i.e*, ICM and ICSOM) and in the *Lentinus tigrinus*-augmented microcosms (*Lt*M and *Lt*SOM) after 60 d at 28°C.

†Data are mean ± standard deviation of two independent experiments; * Multiple pairwise comparisons were performed between row means (P ≤ 0.05); lowercase letters indicate absence of statistically significant differences.

**Figure 6:**
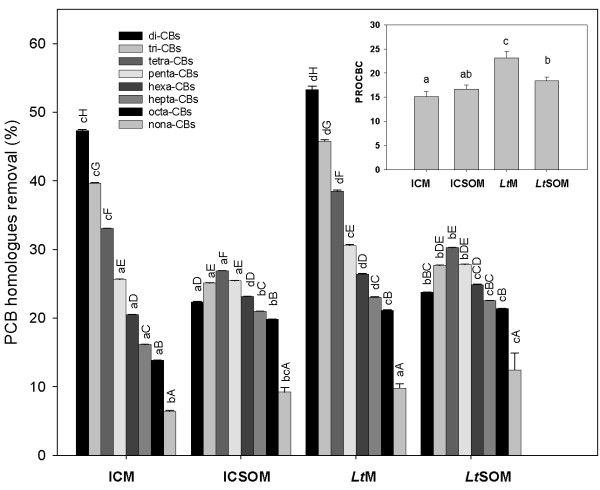
**Percent removal and dechlorination of PCB homologues in myco-augmented microcosms and respective controls**. Removal (%) of PCB homologue groups in the PCB-contaminated soil after bioremediation at 28°C for 60 d in the incubation control and *Lentinus tigrinus *microcosms in the absence (*i.e*., ICM and *Lt*M respectively) and in the presence of 2.5% (w/w) soybean oil (*i.e*., ICSOM and *Lt*SOM). Data are means ± standard deviations (error bars) of two replicated experiments. Multiple pair-wise comparisons were performed by the Tukey test (P < 0.05). Same lower case and upper case letters denote absence of statistical significance between supplementation-dependent changes within the same PCB-homologue group and between PCB-homologue groups for the same treatments, respectively. The overall PCB percent removals were as follows: ICM, 27.8 ± 0.07; ICSOM, 24.5 ± 0.03; *Lt*M, 33.6 ± 0.32 and *Lt*SOM, 26.9 ± 0.06. The inset plot reports the percent release of organic contaminant-bound chloride (PROCBC), calculated according to Brinkman and de Kok [34].

The aforementioned relationship between percent depletion of homologues and respective degree of halogenation was significantly affected by the presence of soybean oil. In both ICSOM and *Lt*SOM, in fact, depletions increased as the number of chlorine substituents increased from 2 to 4 and then tended to decrease for higher chlorinated homologues (Figure 6). However, pair-wise comparisons between homologous microcosms (*i.e*, ICM *vs*. ICSOM and *Lt*M *vs*. *Lt*SOM, respectively) showed that the depletions of low chlorinated congeners were significantly depressed by the presence of SO. For instance, the observed depletions in ICM and *Lt*M of di-CBs (47.3 and 53.3%, respectively) and tri-CBs (39.7 and 45.8%, respectively) drastically dropped in ICSOM and *Lt*SOM to 22.3 and 23.8%, respectively, and to 25.0 and 27.7%, respectively, for the former and the latter homologues. Albeit to a lesser extent, the same trend was observed for tetra- and penta-CBs. Conversely, the presence of SO differentially affected the depletions of hexa- up to octa-CBs in bioaugmented and incubation control microcosms with respect to those observed in their respective non-amended ones. In fact, high chlorinated homologues in *Lt*M and *Lt*SOM were depleted to the same extent; conversely, ICM exhibited lower removal performances than ICSOM.

The possible occurrence of a variety of interfering effects (*i.e*., volatilization, sorption on either soil colloids or microbial biomass) does not necessarily ensure that congener losses determined by gas-chromatography with electron-capture detection be due to actual biodegradation. With this regard, the impact of biosorption phenomena on apparent PCB degradation was not found to be adequately quantified by the use of heat-killed controls [9]. Thus, since chloride removal is known to occur throughout advanced PCB breakdown steps, the release of chloride was used in the present study as a valuable indicator of their degradation [2]. On the basis of the theoretical content of PCB-bound chloride, calculated according to Brinkman and de Kok [34], and on the initial inorganic chloride contents in the microcosms under study, it was possible to assess that a substantial PCB dechlorination had occurred after 60 d incubation. The inset in Figure 6 shows that PROCBC was higher than 15% in all microcosms and that best dechlorination was observed in *Lt*M (*i.e*., 23.2 ± 1.3%) in agreement with the previously mentioned best depletion performances of this microcosm. With regard to the ability of WRB to perform PCB dechlorination, an oxidative chloride removal at the *para*-position was reported for two penta-CBs [11]. In addition, WRB and extracellular oxidases thereof produced, such as laccases, are able to bring about the oxidative dehalogenation of hydroxylated PCBs [10,11]. These intermediates, generated by both the aerobic biphenyl pathway and the intracellular fungal monooxygenases [3], are thus susceptible to oxidation by laccase, the presence of which was mostly detected in *Lt*M and, to a lesser extent, in *Lt*SOM.

The higher contaminant depletion in *Lt*M than in the corresponding incubation control, significant for the majority of PCB homologues, could be due to a significant contribution of the fungus in the degradation process. This can be inferred by the lower densities of cultivable BDB in *Lt*M than ICM and by the time-dependent decrease in *bph *abundances in the former. Moreover, in addition to the reported capability of *L. tigrinus *to degrade PCBs [4], our strain was found to be able to deplete and detoxify chlorobenzoates [22]. However, the absence in *Lt*M of an evident double-*para *recalcitrance effect, related to the resistance of 4,4'-chloro-substituted congeners to fungal degradation and commonly reported for several fungi [5,9,12,33], might suggest that the higher performances of this microcosm than ICM might be ascribed to a cooperative effect of *L. tigrinus *with the resident microbiota. Such a cooperation might involve its above reported degradation ability of CBAs which are known to exert a significant inhibitory action towards biphenyl-degrading bacteria [35]. In addition, the hyphal network of fungi is known to act as a dispersion vector of specialized bacteria, the movement of which is severely limited by the discontinuity of the water paths under unsaturated solid phase conditions [36,37]. An additional indirect contribution of the fungus to the degradation process might stem from its ability to produce EGH activities in soil, the organic matter content of which was far from being negligible. With this regard, hydrolytic exoenzymes significantly contribute to the mobilization of PCBs from soil, by either shifting the sorption equilibrium in the course of soil organic matter (SOM) transformation into dissolved organic matter or by facilitating contaminant diffusion *via *the hydrolase-promoted reduction in rigidity of SOM [38].

With regard to the effect of SO, its presence negatively affected the depletion of low chlorinated congeners, regardless of the use of bioaugmentation. Explanations pertaining to the failure of SO in enhancing PCB removals might be derived from other studies showing that, although MAs promoted PCB pseudo-solubilization, their use led to decreased degradation rates either due to desorption of degrading microorganisms from the matrix or to surfactant-promoted changes in community composition with a decrease in degrader populations [39,40]. The latter hypothesis seems to be supported by our data since this additive exerted a depressive effect on the abundances of *bph *of the upper PCB degradation pathway and on cultivable biphenyl-degrading bacteria in incubation controls. Conversely, the higher depletions of hexa- up to octa-CBs in ICSOM than in ICM cannot be ascribed to the action of aerobic bacteria, that have been shown to be scarcely competent on these high chlorinated congeners. To explain these findings, possible occurrence of anaerobic micro-niches induced by the persistence of SO might not be ruled out. This additive, in fact, is commonly used in field-scale remediation of soils contaminated with chlorinated aliphatic compounds to provide a reactive bio-barrier and to sustain anaerobic dechlorination [41].

## Conclusions

*L. tigrinus *was able to efficiently colonize the heavily and historically contaminated soil leading to an enhancement of the biodiversity of the resident microbiota albeit with a depressive effect on biphenyl-degrading bacteria. However, fungal augmentation led to PCB removal extents (about 34%) after 60 d incubation which were by far higher than other remediation studies conducted under unsaturated solid phase conditions on actual site soils contaminated by Aroclor 1260 [19,29,30]. Although MMS was used as an inoculum carrier or segregated layer in augmented and incubation control microcosms, respectively, its presence proved to be a key factor since in the latter ones a relevantly higher overall PCB depletion than that achieved with a biostimulation treatment of the same soil with biphenyl (4 g kg^-1^) in a solid-phase reactor (27.8 vs. 2.01%, respectively) was observed [19]. The lack of significant effects on PCB recovery in the MMS-amended soil with respect to the non-amended one and the use of this additive in sterilized form ensure that its effects on degradation were not due to either artifacts in contaminant recovery or to the addition of amendant-associated exogenous microbiota, respectively. Regardless of the augmentation, the addition of SO negatively affected the depletion of low chlorinated congeners and thus its use might not be advisable in soils contaminated by technical PCB mixtures such as Aroclor 1242 and Delor 103.

## Methods

### Materials

The historically Aroclor 1260-contaminated soil, kindly supplied by Area SpA (Ravenna, Italy), was homogenized, air-dried, passed through a 2 mm-sieve and then stored at 4°C until used. Its main properties were as follows: real and potential acidity, 7.0 and 6.4, respectively; WHC, 25.7%; total phosphorous, 0.82%; total organic carbon, 2.1%; total organic matter, 3.6%; total nitrogen, 1.2%; sand, 88%; silt, 11%; clay 1%. Both identity and initial concentrations of PCB congeners in incubation control microcosms (please, see below) are reported in Table 1.

### Microorganism and inoculum preparation

*Lentinus tigrinus *CBS 577.79 was maintained at 28°C on potato-dextrose agar (PDA) and sub-cultured every month. Mycelial fragments from 10-d-old PDA-slant cultures were suspended in 5 ml of sterile deionized water and used as inocula for liquid pre-cultures in 500-ml Erlenmeyer flasks containing 95 ml of the following medium (g l^-1^): glucose, 10; yeast extract (Oxoid, Basingstoke, UK), 5. After 96 h incubation at 28°C under orbital shaking (180 rpm), the pre-cultures were centrifuged (4,000 × g, 10 min) and washed with deionized water. The mycelium was homogenized by Ultra-Turrax (IKA Labortechnik, Staufen, Germany) and added with deionized water to yield a biomass concentration of 10 g l^-1 ^which was then used as the inoculum. Milled maize stalks (MMS), the moisture content of which was adjusted to 75%, were placed in 1-l Pyrex bottles covered with Teflon lined stoppers, sterilized in autoclave (121°C, 30 min) and, after cooling, added with 2.0 ml fungal inoculum. Fungal cultures were incubated at 28°C for 7 d under stationary conditions.

### Microcosms preparation

Regardless of the presence of the additive, the PCB-contaminated soil (30 g) was invariably adjusted to a moisture content equal to 65% of its WHC and added over either sterile (120°C, 30 min) non-inoculated MMS or the same fungal-overgrown substrate (7.5 g dry mass) to yield, respectively, the incubation control and *L. tigrinus *microcosms (ICM and *Lt*M, respectively). To prepare the soybean-amended incubation control and *L. tigrinus *microcosms (ICSOM and *Lt*SOM), soil (30 g) underwent nebulization with a previously sterilized (121°C for 15 min) oil-water emulsion (1:1, w/w) to reach an oil concentration of 2.5% (w/w) and then layered over either sterile (120°C, 30 min) non-inoculated MMS or fungal-overgrown substrate (7.5 g dry mass). All experiments were conducted in duplicate at 28°C for 30 and 60 d in 1-l Pyrex bottles covered with Teflon lined stoppers under stationary conditions.

### Extraction of PCBs and analytical procedures

PCBs were extracted from the soil-phase by using a hexane:acetone (1:1, v/v) mixture in a Pressurized Fluid Extraction system (Dionex Corporation, Sunnyvale, CA, USA) operating at 140 atm and 100°C according to the procedure US-EPA-SW-846, Method 3545A. Both qualitative and quantitative PCB analyses were performed with a gas chromatograph (5890 series II), equipped with a HP-5 capillary column (30 m by 0.25 mm) and an electron capture detector (Hewlett-Packard, Palo Alto, CA) as previously described [42]. The depletion of each congener was calculated from the mean of two chromatographic runs. On an average basis, percent residual standard deviation in the quantitation of congeners amounted to 0.24%. Overall degradation activities in each microcosm were determined by both the total weight of residual PCBs obtained by summing the concentrations of each congener and by the summation of contaminants with the same extent of chlorination (homologues). To determine chloride ion contents in incubation controls and bioaugmented microcosms, specimens were added with double distilled water to yield a 20% (w/v) slurry and orbitally shaken (200 rpm) for 2 d at 20°C. Then, the resulting suspensions were centrifuged (11,000 *g*, 15 min) and the supernatants passed through 0.45 *μ*m Minisart syringe filters (Sartorius, Göttingen Germany). The filtrates were then analyzed according to the method of Florrence and Farrar [43]. To determine the percent release of organic contaminant-bound chloride (PROCBC), the differences between chloride contents after 60 d incubation and at start in each microcosm were related to the theoretical PCB-bound chloride calculated according to Brinkman and de Kok [34].

### Bacterial enumeration, mycelial growth and biochemical assays

The concentration of the aerobic heterotrophic cultivable bacterial biomass and that of the biphenyl- or CBA-growing aerobic cultivable bacteria was determined by the plate counting technique described by Fava and Di Gioia [18]. Extraction and subsequent HPLC determination of ergosterol, as a specific index of fungal biomass, were carried out as already reported [23]. Enzymes were extracted from soil samples and subsequently assayed as previously reported [8]. EGH (*i.e*., endo-β-1,4-glucanase, cellobiohydrolase and endo-β-1,4-xylanase), EPO (*i.e*, laccase and Mn-dependent peroxidase) and dehydrogenase activities were determined as reported by Leonardi et al. [8]. All activities were expressed in milli-International Units (mIU), defined as the amount of enzyme producing 1 nmol of product per minute under the assay conditions.

### DNA extraction, PCR amplification and DGGE analyses

Total community DNA was extracted from soil (250 mg) using the Power Soil DNA Extraction Kit (MoBio Laboratories, Carlsbad, CA) following the manufacturer's instruction. Variable regions of 16S and 18S rRNA genes were amplified separately from 10 ng of DNA in a PCR reaction with 0.4 and 0.8 μM of the primers reported by Muyzer et al. [44] and Das et al. [45], respectively, using the illustra*™ *HotStart Master Mix (GE Healthcare, Little Chalfont UK). PCR amplification was performed in a thermal cycler (Bio-Rad Laboratories, Hercules, CA) as previously reported [28]. PCR products from 3 parallel amplifications were pooled, concentrated with a Microcon filter (Millipore, Bedford, MA), separated in 1.5% (w/v) agarose gel and then stained with ethidium bromide.

The INGENYphorU-2 system for DGGE (Ingeny International BV, Goes, NL) was used. Protocol of analysis and estimation of microbial diversity indices (*i.e*., richness, *S*; Shannon-Weaver index, *H*) for each sample were as already reported [28]. An unweighed pair group method with arithmetic means (UPGMA) dendrogram was generated from a similarity matrix based on common band positions between lanes and calculated using the Dice's coefficient [46].

### Quantitative real-time PCR assays

Quantitative real-time PCR (qPCR) was performed on an iCycler IQ (BioRad, Hercules, CA) using the SYBR Green JumpStart™ Taq ReadyMix™ (Sigma, Milan, Italy) following the manufacturer's instruction. The amplification of 16S rRNA genes was performed with 0.05 μM and 0.9 μM of primers 341 F and 534R [44], respectively. The biphenyl dioxygenase (bph) and catechol 2,3-dioxygenase (C230) catabolic genes were amplified using 0.4 μM each of primers BPHF3 and BPHR3 [47] and 0.2 μM each of primers C230F and C230R [48], respectively. Amplification was carried out in a total volume of 25 μl containing 12.5 μl of 2x SYBR Green JumpStart Taq mix, 2.5 μl of each primer and 7.5 μl of template DNA. To avoid PCR amplification problems due to the presence of inhibitors, dilution of environmental DNA samples ranged from 10 to 100 times.

The amplifications were carried out with a first step of 95°C (5 min), followed by 50 cycles of 30 s of denaturation at 95°C, 30 s at 55, 60, and 57°C, for the 16S rRNA, bph and C230 genes, respectively, and 30 s of elongation at 72°C. The final step consisted of 7 min at 72°C. At the end of the qPCR a melting curve analysis was performed by measuring the SYBR Green I signal intensities during a 0.5°C temperature increment every 10 s from 50°C to 95°C. Abundances of target genes in the microcosms investigated were expressed as changes (fold) with respect to their relative zero-timepoint, according to the expression:

(1) $$Fold=2^{\left(Ctx\right)-\left(Cto\right)}$$

where Cto and Ctx are the threshold cycles for the zero and successive time-points, respectively. The threshold cycle (Ct) is the cycle number at which the fluorescence generated within a reaction crosses the threshold. The specificity of the qPCR assays was confirmed by the occurrence of both single melting peaks and unique bands of the expected size on agarose gels.

## Abbreviations

BDB: biphenyl-degrading bacteria; Bph: biphenyl dioxygenase; CBA: chlorobenzoic acids; C230: catechol-2,3-dioxygenase; DGGE: denaturing gradient gel electrophoresis; EGH: extracellular glycosyl hydrolases; *H*: Shannon-Weaver index; ICM: incubation control microcosm; ICSOM: incubation control microcosm with soybean oil; EPO: extracellular phenoloxidases; *Lt*M: *Lentinus tigrinus *microcosm; *Lt*SOM: *Lentinus tigrinus *microcosm with soybean oil; MAs: mobilizing agents; MMS: milled maize stalks; PCBs: polychlorobiphenyls; PROCBC: percent release of organic contaminant-bound chloride; QPCR: quantitative real-time polymerase chain reaction; S: richness; SO: soybean oil; SOM: soil organic matter; WHC: water-holding capacity; WRB: white-rot basidiomycetes.

## Competing interests

The authors declare that they have no competing interests.

## Authors' contributions

FE, GZ, FF, MP and AD have equally contributed to the conception of the experimental design, interpretation of data and to the preparation of the current version of this submission. MG, AN and GS contributed to data acquisition and analysis and have been actively involved in drafting the manuscript and in the approval of its current version. All authors read and approved the final manuscript.
